# Irisin Predicts Poor Clinical Outcomes in Patients with Heart Failure with Preserved Ejection Fraction and Low Levels of N-Terminal Pro-B-Type Natriuretic Peptide

**DOI:** 10.3390/biom14121615

**Published:** 2024-12-17

**Authors:** Tetiana A. Berezina, Oleksandr O. Berezin, Evgen V. Novikov, Michael Lichtenauer, Alexander E. Berezin

**Affiliations:** 1 Department of Internal Medicine and Nephrology, VitaCenter, 69000 Zaporozhye, Ukraine; talexberezina@gmail.com; 2 Luzerner Psychiatrie AG, 4915 St. Urban, Switzerland; lunik.mender@gmail.com; 3 Department of Functional Diagnostics, Shupyk National Healthcare University of Ukraine, 04136 Kyiv, Ukraine; doctornovikov@ukr.net; 4 Department of Internal Medicine II, Division of Cardiology, Paracelsus Medical University, 5020 Salzburg, Austria; m.lichtenauer@salk.at

**Keywords:** heart failure with preserved ejection fraction, clinical outcomes, natriuretic peptide, circulating biomarkers, irisin, visfatin

## Abstract

Background: Despite existing evidence of the high predictive value of natriuretic peptides (NPs) in patients with heart failure (HF), patients treated with guideline-directed therapy who have low or near-normal NP levels are unlikely to be correctly stratified for risk of clinical outcomes. The aim of this study is to detect plausible predictors for poor one-year clinical outcomes in patients with HFpEF and low NT-proBNP treated with in accordance with conventional guidelines. Methods: A total of 337 patients with HF with preserved ejection fraction (HFpEF) who had low levels of N-terminal natriuretic pro-peptide (NT-proBNP) at discharge due to optimal guideline-based therapy were enrolled in the study. The course of the observation was 3 years. Echocardiography and the assessment of conventional hematological and biochemical parameters, including NT-proBNP, tumor necrosis factor-alpha, high-sensitivity C-reactive protein (hs-CRP), adropin, irisin, visfatin, and fetuin-A, were performed at baseline and at the end of the study. Results: Three-year cumulative clinical endpoints (cardiovascular death, myocardial infarction or unstable angina or acute coronary syndrome, worsening HF, sudden cardiac death, or cardiac-related surgery or all-cause death) were detected in 104 patients, whereas 233 did not meet the endpoint. After adjusting for an age ≥ 64 years and a presence of atrial fibrillation, diabetes mellitus, chronic kidney disease (CKD) stages 1–3 and dilated cardiomyopathy, the multivariable Cox regression analysis showed that an irisin level of ≤7.2 ng/mL was an independent predictor of cumulative clinical endpoint. Moreover, patients with levels of irisin > 7.2 ng/mL had a better Kaplan–Meier survival rate than those with a lower serum irisin level (≤7.2 ng/mL). Conclusions: Multivariable analysis showed that an age ≥ 64 years; the presence of atrial fibrillation, diabetes mellitus, CKD stages 1–3 and dilated cardiomyopathy; an LAVI ≥ 39 mL/m^2^; and serum levels of hs-CRP ≥ 6.10 mg/L, irisin ≤ 7.2 ng/mL, and visfatin ≤ 1.1 ng/mL were predictors of poor clinical outcomes in HFpEF with low levels of NT-proBNP. A serum level of irisin ≤ 7.2 ng/mL could emerge as valuable biomarker for predicting long-term prognosis among HFpEF patients with low or near-normal levels of NT-proBNP.

## 1. Introduction

Heart failure (HF) remains a highly prevalent life-threatening condition in older adults that is associated with a high 1-year risk of death and hospitalization, as well as poor functional capacity and quality of life [1]. The median prevalence rate of all HF phenotypes is 11.8% (range 4.7–13.3%), but over the last decade, HF with preserved ejection fraction (HFpEF) has been more common [median prevalence 4.9% (range 3.8–7.4%)] than HF with reduced ejection fraction (HFrEF) [median prevalence 3.3% (range 2.4–5.8%)] [2]. However, HF prevalence, new incidence and survival varied widely in close connection with age, gender, ethnicity, HF phenotype, concomitant comorbidity profile, and certain socio-demographic factors, including affordability of health system resources and guideline-directed medical therapies [3,4,5]. On the other hand, early-to-moderate stages of HFpEF remain more frequently under-recognized than symptomatic HFrEF or HF with mildly reduced ejection fraction (HFmrEF) in everyday practice due to a wide range of comorbidity patterns, including metabolic syndrome, obesity, diabetes mellitus, chronic kidney disease, respiratory and autoimmune diseases [6,7].

Although patients with any HF phenotypes showed a strict similarity in short-term hospitalization rate, the long-term survival rate may vary sufficiently depending on age, HF etiology, comorbidity status and N-terminal brain natriuretic pro-peptide (NT-proBNP) levels [8]. In fact, HF patients with low (<300 ng/L)/near-normal (<125 ng/L) NT-proBNP levels had a better prognosis than those with elevated NT-proBNP levels (>300 ng/L), regardless of HF phenotype [9]. A high proportion of the individuals with any HF phenotype with low NT-proBNP levels exhibits a high incidence of diabetes and obesity. In alignment with this, among of those who reached target levels of NT-proBNP (<1000 ng/L), there were no significant differences in the presence of metabolic comorbidities, but many patients had better clinical status and cardiac performance, including improved left ventricular ejection fraction (LVEF) and quality of life and greater longevity than those with higher levels of NT-proBNP [10,11].

Another aspect concerns patients with HF who were discharged from hospital after decompensation with hemodynamic stability, improved LVEF and low NT-proBNP. More of these individuals were treated with conventional guideline-based therapy, including renin-angiotensin-aldosterone system antagonists, beta-blockers, mineralocorticoid receptor antagonists and sodium-glucose cotransporter-2 (SGLT2i) inhibitors [12]. However, near-normal/low levels of NT-proBNP seem not to be a significant predictive factor for further adverse clinical outcomes, including mortality and HF-related outcomes, when compared with elevated concentrations of this pro-peptide [13]. In fact, there are no evidence-based recommendations for clinical risk assessment in patients with HF with improved LVEF (HFimpEF)/HFpEF with low or near-normal NT-proBNP [14]. Although numerous clinical studies have identified numerous plausible predictors (male sex, left atrial volume index, left ventricular end diastolic dimension, anemia, neutrophil count, the levels of myokines/hepatokines including irisin and adropin, glomerular filtration rate, creatinine, pre-existing kidney failure, atrial fibrillation, diabetes mellitus) for these patients [15,16,17,18,19], reliable predictors corresponding with this clinical presentation remain to be discussed scientifically. Moreover, the predictive utility of metabolic-related factors for HF outcomes, such as adipokines with inflammatory activity, myokines, hepatokines, and inflammatory cytokines, is not still fully recognized [20,21,22].

Indeed, visceral, perivascular and epicardial adipose tissues, the myocardium, skeletal muscles, and the liver are considered endocrine organs that synthesize and release a broad spectrum of cytokines with pro- and anti-inflammatory properties, many of which are directly and indirectly involved in the pathogenesis of adverse cardiac remodeling and are responsible for HF development and progression [23]. Apelin is an anti-inflammatory and angiopoetic adipokine, whose levels are markedly reduced in patients with chronic HF and upregulated after reversible structure cardiac remodeling [24]. Circulating levels of visfatin—a metabolic regulator of oxidative phosphorylation and suppressor of oxidative stress—were found to be significantly lower in patients with HF [25]. Fetuin-A—multifunctional adipokine/hepatokines (known as tissue chaperone) with organoprotective properties—presented a link with liver hypoperfusion in HFrEF and was inversely correlated with exercise tolerance and survival [26]. Patients with HF, especially those with HFrEF and HFmrEF, had sufficiently reduced serum concentrations of adipokine/myokines irisin, which has organ-protective and anti-inflammatory properties, and increased levels of inflammatory cytokines such as tumor necrosis factor-alpha and C-reactive protein [27]. Indeed, the synthesis of irisin—an out-membrane part of fibronectin type III domain-containing 5 protein—may be activated in skeletal muscles, the myocardium and adipose tissue by peroxisome proliferator-activated receptor γ coactivator-1α during physical exercise [28]. Irisin acts by binding to its receptor integrin, αV/β5, to induce the browning of white adipose tissue, maintain glucose homeostasis, maintain bone homeostasis and attenuate cardiac injury. Along with this, irisin may regulate muscle regeneration, promote neurogenesis and angiogenesis, maintain hepatic gluconeogenesis, and ameliorate cardiac injury [29,30]. In numerous pathological conditions, irisin attenuates mitochondrial dysfunction, suppresses oxidative stress, and improves metabolic imbalance and energy expenditure [31]. Irisin levels are inversely associated with the severity of HF, especially with HFrEF, and negatively correlated with myocardial infarct area [31,32]. However, their discriminative abilities in patients with HFpEF with low NT-proBNP have not yet been deeply studied. The aim of this study is to detect plausible predictors for poor one-year clinical outcomes in patients with HFpEF and low NT-proBNP treated with in accordance with conventional guidelines.

## 2. Materials and Methods

### 2.1. Study Population

**Figure 1:**
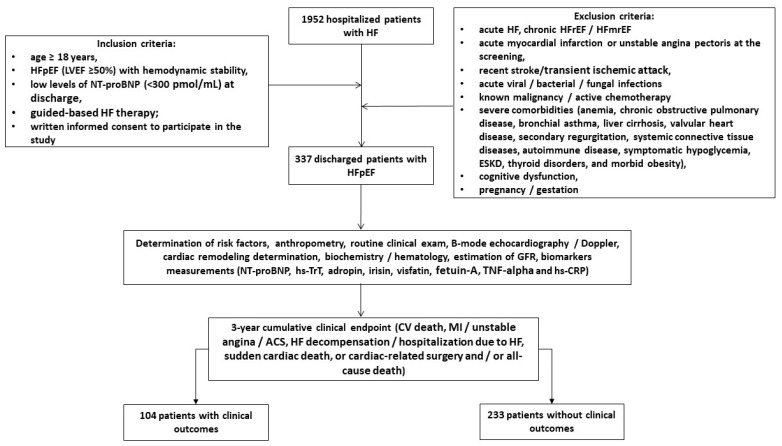
Flowchart of the study design. Abbreviations: ACS, acute coronary syndrome; CV, cardiovascular; ESRD, end-stage renal disease; HF, heart failure; HFpEF, heart failure with preserved ejection fraction; HFrEF, heart failure with reduced ejection fraction; HFmrEF, heart failure with mildly reduced ejection fraction; LVEF, left ventricular ejection fraction; MI, myocardial infarct; TNF-alpha, tumor necrosis factor-alpha; hs-CRP, high-sensitivity C-reactive protein; hs-TrT, high-sensitivity troponin T; NT-proBNP, N-terminal natriuretic pro-peptide.

Using our local database, we pre-screened 1952 patients with HF who were hospitalized for HF progression and had elevated NT-proBNP (≥450 pg/mL) at discharge with a descending trajectory of NT-proBNP during hospital stay. Finally, we enrolled 337 discharged patients according to the following inclusion criteria: age ≥ 18 years, hemodynamic stability and established HFpEF at hospital discharge, low levels of NT-proBNP (<300 pg/mL) at hospital discharge, and written informed consent to participate in the study (Figure 1). The major exclusion criteria included the following: patients with acute HF, HF with reduced (HFrEF) and mildly reduced (HFmrEF) ejection fraction, acute myocardial infarction, unstable angina, recent stroke/transient ischemic attack, acute viral and bacterial infection, known malignancy, active chemotherapy, with severe comorbidities, including end stage renal disease (ESRD), cognitive dysfunction, dementia, pregnancy or gestation. We followed patients for 3 years and divided them into two cohorts depending on the presence of clinical combined outcomes.

### 2.2. Determination of Clinical Outcomes and Follow-Up

We determined a 3-year cumulative clinical endpoint that included CV death, myocardial infarction or unstable angina or acute coronary syndrome, HF decompensation or hospitalization due to HF, sudden cardiac death, or cardiac-related surgery and/or all-cause death. To detect the cumulative clinical endpoint, we conducted direct interviews with patients and their relatives, contacted general practitioners, and reviewed databases, discharge and autopsy reports. Data were collected via clinic visits at baseline (at discharge from the hospital) and during the 36 months following the start of the study.

### 2.3. Echocardiography Examination

In the study, all patents underwent a routine transthoracic B-mode and Doppler ultrasound examination, which was performed by experienced echo cardiographer in apical two- and four-chamber views using a GE Healthcare Vivid E95 scanner (General Electric Company, Horton, Norway). The conventional hemodynamic parameters included the left ventricular ejection fraction (LVEF) using Simpson’s method, the left ventricular end-diastolic (LVEDV) and end-systolic (LVESV) volumes, the left atrial volume index (LAVI), early diastolic blood filling (E), and the mean longitudinal strain ratio (e′), which were evaluated according to the 2018 guidelines of the American Society of Echocardiography [33]. The estimated E/e′ ratio was expressed as the ratio of the E-wave velocity to the average of the medial and lateral e’ velocities. After the acquisition of high-quality echocardiographic data during at least three cardiac cycles, LV GLS was obtained by 2D speckle-tracking image analysis. The data were stored in the DICOM format for subsequent analysis. The left ventricular mass index (LVMI) was defined as ≥95 g/m^2^ in women or ≥115 g/m^2^ in men [33].

### 2.4. Blood Sampling

Blood samples were obtained from all participants in fasting condition and collected in BD Vacutainer Serum Plus tubes. After centrifugation at 3000 rpm for 10 min, the supernatant was collected and stored at −70 °C until analysis.

### 2.5. Biomarkers Assessment

Conventional hematological and biochemical parameters were determined with a Roche P800 analyzer (Basel, Switzerland) in the local laboratory of the Vita Centre (Zaporozhye, Ukraine). Data on routine blood indices, including glucose, total cholesterol, triglycerides, high-density lipoprotein (HDL-C) and low-density lipoprotein (LDL-C) cholesterol, serum uric acid, and serum creatinine, were recorded. In addition, we measured circulating biomarkers (NT-proBNP, tumor necrosis factor-alpha [TNF-alpha], high-sensitivity C-reactive protein [hs-CRP], adropin, irisin, visfatin, fetuin-A) in serum using ELISA kits (Elabscience, Houston, TX, USA) in accordance with the instructions provided by the manufacturer at the initial baseline measurement and at the end of the study. The data obtained from the ELISA analysis were subjected to a standard curve-based evaluation. Each sample was analyzed in duplicate, and the mean value was employed for the final analysis. Both intra- and inter-assay coefficients of variability for each marker were <10%.

### 2.6. Glomerular Filtration Rate Estimation

A conventional CKD-EPI formula was to estimate the glomerular filtration rate (eGFR) [34].

### 2.7. Statistics

All statistical analyses were conducted using SPSS 11.0 for Windows and Graph Pad Prism, version 9 (Graph Pad Software, San Diego, CA, USA). A Kolmogorov–Smirnov test was used to determine whether data were normally distributed. All continues variables were expressed as mean ± standard deviation [SD] median and interquartile range [IQR] depending on whether the data were normally distributed, whereas categorical variables were presented as number (n) and percentage (%). Categorical variables were compared using the chi-squared test. The Mann–Whitey U test was used to compare differences in continuous variables between cohorts with and without combined clinical events. Receiver operating characteristic (ROC) curves were used to calculate the cutoff values of irisin for predicting combined clinical events. To determine the optimal cutoff value for the predictors, Youden’s index (sensitivity + specificity − 1) was used. Univariate and multivariate Cox proportional hazard models were constructed to predict the independent prognostic factors for the clinical endpoint. Significant variables (*p* < 0.05) comparing cohorts with and without cumulative endpoints were entered into univariate Cox regression analysis, and those retaining statistical significance (*p* < 0.05) were entered into the multivariate Cox proportional hazards model. The odds ratio (OR) and 95% confidence interval (CI) were reported for each predictor variable. The Kaplan–Meier method was used to compare the survival rates, and survival curves were plotted. A *p*-value of less than 0.05 was considered to indicate a statistically significant difference.

## 3. Results

### 3.1. Baseline Clinical Characteristics

A total of 337 patients with HFpEF, New York Heart Association class II (*n* = 133)/III (*n* = 204), and low NT-proBNP at discharge were enrolled in the study and divided into two cohorts depending on the presence of a 3-year cumulative endpoint (*n* = 104) or an absent of it (*n* = 233). The follow-up time was 3 years. The clinical characteristics of the patients are outlined in Table 1.

Patients in the entire group were respectively young (a mean age of 61 years), mainly male, and had a low presentation frequency of atrial fibrillation, diabetes mellitus, abdominal obesity, and chronic kidney disease. However, there was a high presence of hypertension, left ventricular hypertrophy and the NYHA III functional class. They were well-treated with antagonists of renin-angiotensin-aldosterone system (ACE inhibitors, or angiotensin-II receptor blockers or angiotensin receptor-neprilysin inhibitors), beta-blockers, mineralocorticoid receptor antagonists and sodium–glucose cotransporter-2 inhibitors according to guidelines, whereas only 58% of the patients had a need for maintenance loop diuretics at discharge.

There were no significant differences between the two cohorts with respect to sex, body mass index, anthropometric parameters, presence of dyslipidemia, hypertension, stable coronary artery disease, smoking, abdominal obesity, systolic and diastolic blood pressure, left ventricular dimensions, LVEF, LVMI, E/e′, GLS, eGFR, lipid profile, glucose levels, TNF-alpha, NT-proBNP, adropin, visfatin, fetuin-A, or hs-TrT. Patients in the cumulative clinical endpoint cohort were older; more likely to have atrial fibrillation and diabetes mellitus; be in CKD stages 1–3; and have dilated cardiomyopathy, higher LAVI and lower irisin levels than those in the free endpoint cohort. The patients in the cumulative clinical endpoint cohort tended to be treated frequently with angiotensin-II receptor blockers, anticoagulants, metformin when compared with those who had no clinical endpoint.

### 3.2. Receiver Operating Characteristic Curve Analysis for Predictive Factors of Cumulative Clinical Endpoint

ROC curve analysis was performed to determine the optimal cutoff for possible predictors of clinical outcomes, shown in Table 2. We identified the following factors: age ≥ 64 years, hemoglobin < 125 g/L, body mass index ≥ 27.5 kg/m^2^, LAVI ≥ 39 mL/m^2^, hs-CRP ≥ 6.10 mg/L irisin and visfatin ≤ 7.2 ng/ and ≤1.1 ng/mL, respectively.

### 3.3. Predictive Factors for 3-Year Cumulative Clinical Endpoint: Unadjusted and Adjusted for Multivariate Cox Proportional Hazard Models

A Cox proportional hazards model was constructed for the assessment of independent predictors of the 3-year cumulative clinical endpoint (Table 3).

After adjusting for an age ≥ 64 years and the presence of atrial fibrillation, diabetes mellitus, CKD stages 1–3 and dilated cardiomyopathy (Model 2), the multivariable Cox regression analysis revealed that a level of irisin ≤ 7.2 ng/mL was an independent predictor of the cumulative clinical endpoint.

### 3.4. Kaplan–Meier Curves Survival Analysis

**Figure 2:**
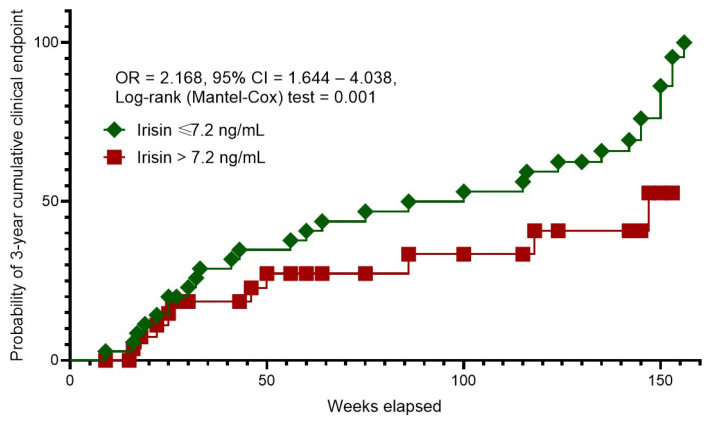
Kaplan–Meier curves for 3-year cumulative clinical endpoint. Abbreviations: OR, odds ratio; CI, confidence interval.

The Kaplan–Meier curves revealed different probability rates of 3-year cumulative clinical endpoint between patients with HFpEF and serum irisin levels of ≤7.2 ng/mL and >7.2 ng/mL (Figure 2). Of note, patients with irisin levels > 7.2 ng/mL had a sufficient benefit in terms of survival than those with serum irisin levels ≤ 7.2 ng/mL.

## 4. Discussion

Only HFpEF patients who responded to optimal guided-based therapy and at discharge gave the impression of adequately compensated low-risk patients were included in this study. We identified an age ≥ 64 years, the presence of atrial fibrillation, diabetes mellitus, CKD stages 1–3 and dilated cardiomyopathy, an LAVI ≥ 39 mL/m^2^, and serum levels of hs-CRP ≥ 6.10 mg/L, irisin ≤ 7.2 ng/mL and visfatin ≤ 1.1 ng/mL as predictors of poor clinical outcome in HFpEF patients treated with guideline-based optimal therapy with target level of NT-proBNP less than 300 pmol/mL. After adjusting for concomitant comorbidities and age, serum levels of irisin ≤ 7.2 ng/mL remained an independent predictive factor for the 3-year cumulative clinical endpoint. Moreover, the individuals with circulating irisin > 7.2 ng/mL exerted a significant superiority in survival rate when compared with those with lower irisin levels (≤7.2 ng/mL). These findings offer new perspectives in the stratification of patients with HFpEF who have achieved target NT-proBNP levels as a result of conventional management and/or concomitant comorbidities such as obesity.

The hypothesis is as follows: this is an apparently low risk that does not reflect the population level of risk in people without HFpEF, and these patients are under-screened individuals for whom the risk of adverse outcomes is underestimated. Irisin is likely to be the molecule that overcomes the limitations of natriuretic peptides in identifying patients at residual risk in the context of optimal treatment.

Previous studies and systematic reviews highlighted the plausible prognostic role of BNP and NT-proBNP in predicting adverse clinical outcomes, including all-cause mortality, cardiovascular events, and hospitalization in HFpEF patients, [13,35,36]. However, the predictive role of NPs in HFpEF seem to be controversial, because low levels of NT-proBNP often leave the risk of HF progression underestimated [37]. Meanwhile, patients hospitalized with HFpEF often have low NT-proBNP levels, which was associated with age < 65 years, ischemic etiology, obesity, higher LVEF, eGFR > 80 mL/kg/1.73 m^2^, and a lower NYHA class, whereas chronic kidney disease and diabetes mellitus were associated with elevated levels of NT-proBNP [9,38,39]. Moreover, clinical status was similarly impaired in HF patients with lower and higher NT-proBNP levels [40]. Azzo JD et al. (2024) [41] reported that higher levels of NT-proBNP were more associated with inflammation and fibrosis than low concentrations of the biomarker. On the other hand, the elevation of NT-proBNP in serial measurements reflected a dynamic change in risk for HF events and death, whereas stable levels of NT-proBNP were related to a lower risk of incident HF and mortality [42]. Thus, the risk of patients with low NP levels remains often out of the zone of interest or is only used as a reference level when comparing to higher values of NT-proBNP. The use of cardiac troponins as an alternative biomarker for risk stratification in a patient with low NT-proBNP levels also remains inadequate because a positive troponin test improves the predictive and diagnostic value of natriuretic peptides only when their concentrations are high [43]. In this context, the search for new biomarkers with independent predictive value for adverse clinical events in HFpEF and low NT-proBNP remains very relevant.

We hypothesized that the comorbidity signature may not only contribute to adverse cardiac remodeling but may also be associated with a distinct cytokine profile reflecting the metabolic homeostasis of distant organs, such as skeletal muscle, adipose tissue, and liver. Although these organs synthesize and secrete a wide range of metabolically active molecules involved in the development and progression of HF, their discriminative properties for HF, particularly in people with HFpEF, have not been established. In this study, we established that apart from older age and known concomitant comorbidities such as atrial fibrillation, diabetes mellitus, CKD stages 1–3 and dilated cardiomyopathy, altered levels of hs-CRP, visfatin and irisin (which belong to the adipokines/myokines) were predictors for cumulative clinical endpoint for 3 years in patients with HFpEF and low levels of NT-proBNP. In fact, an irisin level ≤ 7.2 ng/mL alone was found to be an independent predictor of adverse clinical outcomes for patients with HFpEF after adjusting for age and concomitant comorbidities.

Previous studies have shown that irisin can be synthesized and secreted not only by skeletal muscle but also by the myocardium to maintain energy homeostasis [44]. The alteration of irisin levels in HF is considered a possible mechanism of skeletal muscle metabolic remodeling, cardiac hypertrophy and the persistence of a clinical sign such as fatigue [45]. Moreover, in chronic HFpEF patients rather than those with HFmrEF and HFrEF, low levels of irisin exerted predictive potency for adverse outcomes [15,46,47]. Since irisin has shown an inverse correlation with inflammatory biomarkers such as CRP and TNF-alpha, and LDL in previous studies, it was hypothesized that one of the possible molecular mechanisms for the negative impact of altered levels of irisin on prognosis is excessive inflammatory response, oxidative stress/damage and mitochondrial dysfunction due to activation of apoptosis/pyroptosis and autophagy via irisin precursor fibronectin type III domain-containing protein 5 and the AKT/GSK3β/FYN/Nrf2 axis in an mTOR-independent manner [48,49]. In this context, altered irisin regulation links adverse cardiac remodeling and skeletal muscle dysfunction with metabolic comorbidities such as diabetes mellitus, obesity and metabolic syndrome [50,51]. In addition, irisin may have the ability to modify cellular plasticity through alternative signaling pathways such as cAMP/PKA/CREB and Wnt signaling [52]. In this context, the molecular targets of irisin may correspond to those of lysine β-hydroxybutyrylation (Kbhb), a regulator of post-translational modification [53]. This may suggest that irisin acting through the Kbhb-related enhancement of mitochondrial enzyme activity such as glutamate oxaloacetate transaminase is likely to be crucial for preventing mitochondrial dysfunction and the progression of structural cardiac remodeling. Therefore, irisin along with a product of histone Kbhb activation β-hydroxybutyrate—the main component of the ketone body—may induce the ketogenesis, which, acting through SIRT3-dependent pathways, ensures adaptive metabolic changes in mitochondria [54]. Finally, the cardioprotective effects (anti-arrhythmic and anti-ischemic effects) of several agents, which are included in optimal guideline-based HF therapy, such as sodium-glucose co-transporter 2 inhibitors, can be mediated by restoring circulating irisin levels [15,55,56,57].

However, the predictive value of irisin for distant events in patients with HFpEF requires further explanation. An indirect effect of irisin deficiency on the number of cardiovascular events through the progression of microvascular inflammation and impaired myocardial perfusion cannot be excluded. This suggestion seems to be a reason for further investigations.

## 5. Study Limitations

The study has several limitations. The first limitation is the lack of data regarding the trajectory of biological markers during the observational period in connection with the rate of adverse clinical outcomes. However, we did not investigate the quality of life of the patients and its association with the changes in irisin levels. Finally, we did not compare the discriminative value of irisin with previously validated risk scores. However, we do not believe that these limitations will have an impact on the interpretation of the results.

## 6. Conclusions

Multivariable analysis showed that an age ≥ 64 years; the presence of atrial fibrillation, diabetes mellitus, CKD stages 1–3 and dilated cardiomyopathy; an LAVI ≥ 39 mL/m^2^; and serum levels of hs-CRP ≥ 6.10 mg/L, irisin ≤ 7.2 ng/mL and visfatin ≤ 1.1 ng/mL were predictors of poor clinical outcome in HFpEF. A serum level of irisin ≤ 7.2 ng/mL could emerge as valuable biomarker for predicting long-term prognosis among HFpEF patients with low or near-normal levels of NT-proBNP.

## Figures and Tables

**Table 1:** Basic characteristics of the patients involved in this study.

Variables	Entire Group Patients with Low-NT-proBNP (*n* = 337)	Patients with Clinical Endpoint (*n* = 104)	Patients Without Clinical Endpoint (*n* = 233)	*p* Value
Age (years)	61 (51–73)	65 (54–75)	56 (49–65)	0.040
Male (*n* (%))	172 (51.0)	57 (54.8)	115 (49.4)	0.728
BMI (kg/m^2^)	26.76 ± 6.10	27.10 ± 5.70	25.80 ± 5.10	0.640
Waist circumference (cm)	97.10 ± 3.90	98.10 ± 4.10	96.40 ± 4.20	0.520
WHR (units)	0.89 ± 0.12	0.90 ± 0.10	0.87 ± 0.09	0.430
Dyslipidemia (*n* (%))	203 (60.2)	67 (64.2)	136 (58.4)	0.064
Hypertension (*n* (%))	285 (84.6)	88 (84.6)	197 (84.5)	0.832
Stable CAD (*n* (%))	113 (33.5)	37 (35.6)	76 (32.6)	0.472
Dilated CMP, (*n* (%))	18 (5.3)	11 (10.6)	7 (3.0)	0.010
Atrial fibrillation (*n* (%))	61 (18.1)	22 (21.2)	39 (16.7)	0.020
Smoking (*n* (%))	132 (39.2)	41 (39.4)	91 (39.0)	0.850
Abdominal obesity (*n* (%))	95 (28.2)	30 (28.8)	65 (27.9)	0.720
Diabetes mellitus, (*n* (%))	106 (31.5)	43 (41.3)	63 (27.0)	0.012
LVH (*n* (%))	246 (73.0)	78 (75.0)	168 (72.1)	0.074
CKD stages 1–3 (*n* (%))	72 (21.3)	29 (27.9)	43 (18.5)	0.044
New York Heart Association class II	133 (39.5)	40 (38.5)	93 (39.9)	0.662
New York Heart Association class III	204 (60.5)	64 (61.6)	140 (60.1)	0.664
Systolic BP (mm Hg)	138 ± 9	137± 10	138 ± 9	0.810
Diastolic BP (mm Hg)	83 ± 7	86 ± 8	82 ± 6	0.720
LVEDV (mL)	152 (146–163)	156 (141–171)	153 (144–166)	0.760
LVESV (mL)	70 (62–79)	73 (65–89)	69 (60–76)	0.062
LVEF (%)	54 (51–58)	53 (51–55)	54 (51–58)	0.158
LVMI (g/m^2^)	140 ± 12	143 ± 13	138 ± 10	0.161
LAVI (mL/m^2^)	39 (35–45)	42 (37–48)	37 (35–40)	0.044
E/e′ (units)	15 ± 5	17 ± 4	13 ± 5	0.686
GLS (%)	−15.2 (−13.1; −17.7)	−15.7 (−13.2; −18.1)	−14.5 (−12.5; −16.2)	0.224
eGFR (mL/min/1.73 m^2^)	81 (53–102)	75 (49–95)	89 (50–116)	0.216
Fasting glucose (mmol/L)	4.92 ± 1.1	5.11 ± 1.2	4.84 ± 1.0	0.860
Creatinine (µmol/L)	106.5 ± 22.7	113.4 ± 19.5	99.3 ± 21.4	0.620
SUA (mcmol/L)	332 ± 116	345 ± 85	316 ± 105	0.162
Total cholesterol (mmol/L)	5.87 ± 1.30	5.92 ± 1.24	5.76 ± 1.18	0.226
HDL-C (mmol/L)	0.98 ± 0.23	0.96 ± 0.19	0.99 ± 0.20	0.186
LDL-C (mmol/L)	3.87± 0.24	3.90 ± 0.22	3.85± 0.21	0.650
Triglycerides (mmol/L)	2.26 ± 0.19	2.30 ± 0.17	2.24 ± 0.15	0.440
hs-CRP (mg/L)	5.26 (2.54–8.10)	6.02 (3.10–9.17)	4.46 (2.29–6.76)	0.050
TNF-alpha (pg/mL)	3.11 (2.10–4.22)	3.44 (1.99–4.85)	2.82 (1.80–3.93)	0.070
NT-proBNP (pmol/mL)	198 (115–286)	217 (137–295)	180 (113–253)	0.142
Adropin (ng/mL)	3.62 (2.10–5.11)	3.89 (2.45–5.28)	3.38 (1.98–4.81)	0.244
Irisin (ng/mL)	8.25 (6.30–10.56)	7.01 (5.80–8.49)	9.45 (6.71–11.80)	0.001
Visfatin (ng/mL)	1.32 (0.87–1.79)	1.21 (0.80–1.65)	1.50 (0.89–2.08)	0.050
Fetuin-A (μg/mL)	67.5 (51.1–85.9)	58.6 (43.4–74.2)	75.8 (58.2–92.5)	0.064
hs-TnT, ng/mL	0.05 (0.012–0.114)	0.05 (0.011–0.123)	0.04 (0.008–0.109)	0.688
ACEIs (*n* (%))	252 (74.7)	72 (69.2)	180 (77.3)	0.576
Angiotensin-II receptor blockers (*n* (%))	38 (11.3)	20 (19.2)	18 (7.70)	0.042
ARNI, (*n* (%))	47 (13.9)	12 (11.5)	35 (15.0)	0.352
Beta-blockers (*n* (%))	317 (94.0)	97 (93.3)	220 (94.4)	0.788
Ivabradine (*n* (%))	34 (10.1)	10 (9.6)	24 (10.3)	0.810
Calcium channel blockers (*n* (%))	83 (24.6)	23 (22.1)	60 (25.8)	0.760
Loop and thiazide-like diuretics (*n* (%))	196 (58.2)	64 (61.5)	132 (56.7)	0.064
Antiplatelet agents (*n* (%))	283 (84.0)	81 (77.9)	202 (86.7)	0.052
Anticoagulants (*n* (%))	61 (18.1)	22 (21.2)	39 (16.7)	0.020
Metformin (*n* (%))	95 (28.2)	37 (35.6)	58 (24.9)	0.010
DPP-4 inhibitors (*n* (%))	11 (3.3)	4 (3.8)	7 (3.0)	0.860
GLP-1 receptor agonists (*n* (%))	13 (3.9)	3 (2.9)	10 (4.3)	0.226
SGLT2 inhibitors (*n* (%))	332 (98.5)	101 (97.1)	231 (99.1)	0.884
Statins (*n* (%))	301 (89.3)	92 (88.4)	209 (89.7)	0.870

Notes: Variables are given as Ms ± SDs or Ms (25–75% IQRs). The Chi-square test was used to compare categorical variables. The Mann–Whitney U test, and Chi-square test were used to compare continuous variables between cohorts. LVH was detected when LVMI ≥ 95 g/m^2^ in women or ≥115 g/m^2^ in men. Abbreviations: ACEIs, angiotensin converting enzyme inhibitors; ARNI, angiotensin receptor-neprilysin inhibitors; BMI, body mass index; CAD, coronary artery disease; CMP, cardiomyopathy; CKD, chronic kidney disease; DPP-4, dipeptidyl peptidase-4; eGFR, estimated glomerular filtration rate; E/e′, early diastolic blood filling to longitudinal strain ratio; GLS, global longitudinal strain; GLP-1, glucagon-like peptide-1; HDL-C, high-density lipoprotein cholesterol; hs-CRP, high-sensitivity C-reactive protein; LAVI, left atrial volume index; LDL-C, low-density lipoprotein cholesterol; LVH, left ventricular hypertrophy; LVEDV, left ventricular end-diastolic volume; LVESV, left ventricular end-systolic volume; LVEF, left ventricular ejection fraction; LVMI, left ventricle mass index; NT-proBNP, N-terminal natriuretic pro-peptide; SGLT2, sodium–glucose cotransporter-2; SUA, serum uric acid; TNF-alpha, tumor necrosis factor-alpha; WHR, waist-to-hip ratio.

**Table 2:** Receiver operating characteristic (ROC) curve analysis for predictive factors of 3-year cumulative clinical outcome.

Variables	AUC	95% CI	*p* value	Cutoff	Se, %	Sp, %
Age	0.726	0.712–0.740	0.001	64 years	76.2	74.5
Hb	0.620	0.488–0.749	0.062	125 g/L	63.1	60.3
BMI	0.556	0.472–0.639	0.124	27.5 kg/m^2^	56.8	52.4
LAVI	0.771	0.723–0.835	0.001	39 mL/m^2^	78.5	82.0
hs-CRP	0.755	0.695–0.819	0.001	6.10 mg/L	77.3	80.8
Irisin	0.868	0.799–0.948	0.0001	7.2 ng/mL	83.4	86.3
Visfatin	0.757	0.733–0.787	0.001	1.1 ng/mL	80.5	81.9

Abbreviations: AUC, area under curve; CI, confidence interval; Se, sensitivity; Sp, specificity; CAD, coronary artery disease; Hb, hemoglobin; BMI, body mass index; hs-CRP, high-sensitivity C-reactive protein; LAVI, left atrial volume index.

**Table 3:** Cox regression analysis for predictive factors of cumulative clinical endpoint.

Predictive Factors	Model 1		Model 2	
	HR (95% CI)	*p* Value	HR (95% CI)	*p* Value
Age ≥ 64 years	1.327 (1.064–1.673)	0.026	-	
BMI ≥ 27.5 kg/m^2^	1.042 (1.001–1.106)	0.134	-	
Hb ≤ 125 g/L	1.071 (0.993–1.185)	0.572	-	
CAD (presence vs. absent)	1.050 (0.971–1.143)	0.622	-	
AF (presence vs. absent)	2.035 (1.313–3.184)	0.012	-	
Dilated CMP (presence vs. absent)	1.614 (1.151–2.120)	0.047	-	
DM (presence vs absent)	1.226 (1.115–1.338)	0.048	-	
CKD stages 1–3 (presence vs. absent)	1.116 (1.002–1.237)	0.050	-	
LAVI ≥ 39 mL/m^2^	1.408 (1.122–1.806)	0.048	1.206 (0.966–1.437)	0.523
hs-CRP ≥ 6.10 mg/L	1.183 (1.106–1.450)	0.042	1.042 (1.006–1.092)	0.121
Irisin ≤ 7.2 ng/mL	1.415 (1.211–1.644)	0.042	1.386 (1.254–1.5904)	0.044
Visfatin ≤ 1.1 ng/mL	1.190 (1.102–1.276)	0.049	1.121 (1.003–1.220)	0.068

Notes: Model 1: unadjusted rough model; Model 2: model adjusted for age ≥ 64 years and a presence of concomitant conditions (atrial fibrillation, diabetes mellitus, CKD stages 1–3 and dilated cardiomyopathy). Abbreviations: AF, atrial fibrillation; CAD, coronary artery disease; BMI, body mass index; Hb, hemoglobin; CMP, cardiomyopathy; CI, confidence interval; DM, diabetes mellitus; HR, hazard ratio.

## Data Availability

The data presented in this study are available on request from the corresponding author due to privacy restrictions.
